# Process optimization and technoeconomic environmental assessment of biofuel produced by solar powered microwave pyrolysis

**DOI:** 10.1038/s41598-022-16171-w

**Published:** 2022-07-22

**Authors:** Ahmed Elsayed Mahmoud Fodah, Taha Abdelfattah Mohammed Abdelwahab

**Affiliations:** College of Agricultural Engineering, Al-Azhar University, Cairo, 11765 Egypt

**Keywords:** Biotechnology, Environmental sciences, Energy science and technology

## Abstract

Microwave pyrolysis of corn stover has been optimized by Response surface methodology under different microwave power (500, 700, and 900 W) and three ratios of activated carbon additive (10, 15, and 20%) for obtaining maximum bio-oil yield followed by biochar. The optimal result has been evaluated and the environmental and techno-economic impacts of using solar-powered microwave heating have been tested. The optimal pyrolysis condition found to be 700 W microwave power and 10% of activated carbon. The yields of both bio-oil and biochar were about 74 wt% under optimal condition. The higher heat values of 26 MJ/kg and 16 MJ/kg were respectively achieved for biochar and bio-oil. The major components of bio-oil were hydrocarbons (36%) and phenols (28%) with low oxygen-containing compounds (2%) and acids (2%). Using the solar-powered system, 20,549 tonnes of CO_2_ can be mitigated over the lifetime of the set-up, resulting in USD 51,373 in carbon credit earnings, compared to 16,875 tonnes of CO_2_ mitigation and USD 42,167 in carbon credit earnings from a grid electricity system. The payback periods for solar-powered and grid-connected electrical systems are estimated to be 1.6 and 0.5 years, respectively, based on biochar and bio-oil income of USD 39,700 and USD 45,400.

## Introduction

Valorization of underutilized agricultural residues represents a significant challenge in the context of their safe disposal and the potential recovery of energy in an environmentally sustainable manner. About 40 million tonnes of crop residue are generated every year in Egypt^1,2^. Crop residues are mostly burned in the field. However, around 60% of the gross residue generated remains surplus^1–3^, which becomes a great concern from the environmental point of view. Such a sustainable resource of bioenergy in Egypt (crop residues) needs to be properly utilized to strengthen the poor socio-economic conditions of the crop growers^4^. One such attempt is to derive biofuel and chemicals from underutilized crop residues in a sustainable manner. This option would ultimately assist to earn extra income and reduce environmental pollution by disposing of neglected and surplus agro-residues safely and effectively^4^. One of the most important crop residues in Egypt and across the world is corn stover (CS). The annual production of CS was approximately 7.2 Tg (Tera-gram) in Egypt^3^. Therefore, CS has been selected as a feedstock in this study because it can be considered a sustainable source of bioenergy.

Among the two usual processes, i.e., biological and thermochemical, for conversion of biomass into energy, the latter is preferable to convert the biomass into more useful products^5,6^. Pyrolysis is the rapid thermal decomposition of biomass into pyrolytic products such as biochar, bio-oil, and gas in the absence of oxygen^4,7^, with the goal of recovering both its chemical and energy value with high efficiency and low emissions^5^.

Because of their easy storability and transportability, bio-oil and biochar produced by the pyrolysis process have more applications, both at the user and commercial levels, than pyrolytic gas (syngas)^6–8^. Biochar, a solid biofuel having a higher carbon content, offers many applications in day-to-day life because of its environmental friendliness^9–11^. Its applications are mostly in domestic cooking, boilers, production of activated carbon, absorbing microwaves, preparing nanotubes, conditioning the soil, etc. It is, therefore, easy to trade among the farming community^12,13^. Likewise, bio-oil, one of the pyrolytic products from the thermochemical decomposition of biomass, is a dark brown and viscous organic liquid that offers several utilizations due to the presence of various chemical compounds in it^14,15^. The availability of a number of chemical compounds, in bio-oil, increases its use as an alternate fossil fuel along with its enhanced utilization in the biochemical, packaging, and pharmaceutical industries^6,16^.

Microwave-assisted pyrolysis of biomass (MAP) in recent years has been considered as an emerging pyrolysis technology for saving time, energy, achieving higher heating efficiency, precise control over the process, and a higher yield of the desired products compared to conventional pyrolysis (CP)^17,18^. One difficulty, however, commonly encountered during MAP is the low capacity of microwave absorption by the biomass feedstock due to its poor dielectric properties^19^. Thus, in the MAP process, several methods have been investigated to improve the quality of both bio-oil and biochar. One of the most effective methods is using the additives in the MAP process^8,20,21^. Carbon-based additives are most effective in microwave pyrolysis, thus activated carbon (AC) from the process itself may be considered as an effective additive to improve the heating process and quality of the products^8–25^.

Based on the literature, most of the MAP systems emphasize batch lab-scale with little attention to scaling-up the process in adopting the technology among the producers to make it economically viable^26^. In the agricultural sector, where a huge quantity of agro-residues are generated annually, the scaling-up of the MAP plants may be considered economically viable^27^. However, the initial high operating costs, as well as the economic benefits, are the most important aspects for crop growers to adopt this technology in a scale-up system.

One of the major constraints is the reliability the availability of electrical power for operating the system in rural and off-grid areas. Moreover, increased use of grid-electricity results in the indirect emission of harmful pollutants because its generation is mostly from coal-based thermal power plants, where the burning of coal produces greenhouse gases into the environment. Therefore, the integration of other sources of renewable energy, especially solar energy, as an input power for the MAP process has been found to be more feasible^28,29^.

**Figure 1:**
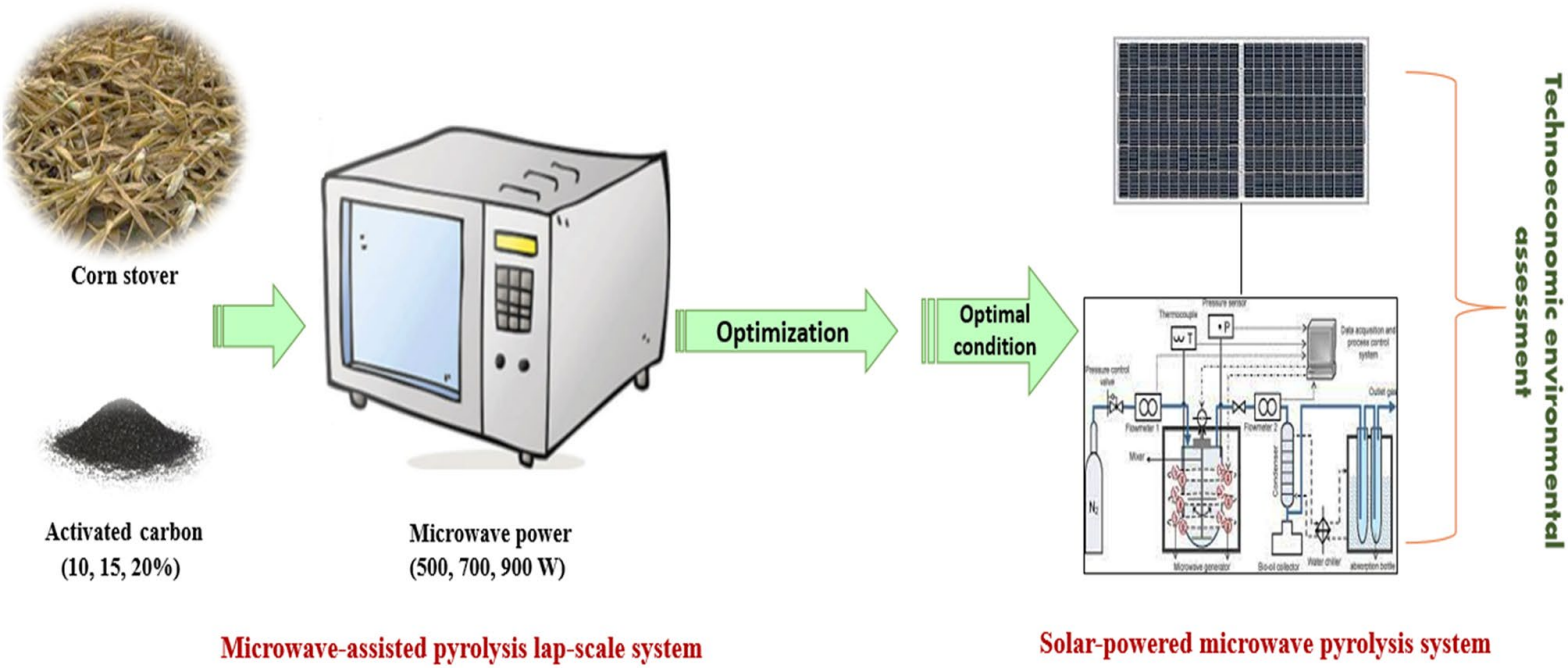
The experimental scheme of study.

From the above discussions, an attempt has therefore been made in this study to provide appropriate insight for valorizing one of the most underutilized agro-residues (i.e., CS) in an agrarian society from economical, environmental, and societal standpoints. Efforts have also been made to experimentally investigate the potential of extracting more useful and value-added products such as bio-oil and biochar from raw CS in a convenient manner by following MAP, integrated with reliable solar PV electricity, with the goals of safely disposing of them, mitigating greenhouse gas emissions by preventing improper uses, and creating avenues for earning extra income, particularly among resource poor crop growers. Therefore, the integration of the MAP process with solar PV power for producing biofuel and value-added products is cost-effective and environmentally sustainable. The process was first optimized to determine the optimal condition of the process with the aim of maximum bio-oil yield followed by biochar (desirable products of the study). Then the products under optimal conditions have been evaluated (quantity and quality). Techno-economic analysis for a 0.75 tonne/h capacity pyrolysis facility has been done based on our experimental investigation. Also, the techno-economic and environmental assessment of the process in the scale-up system using grid electricity and solar PV have been evaluated. Figure 1 represents the experimental scheme of study.

## Materials and methods

### Biomass feedstock and pyrolysis additives

Corn stover (CS) was collected from corn field after the corn crop was harvested. The CS was sun-dried until the excess moisture was removed, then mechanically shredded to reduce the particle size approximately of 10 mm. Table 1 shows the results of the investigation into the CS characterizations. The additive used in this study was activated carbon (AC) in granular form with particle sizes of 2 mm, specific surface area of 1050 m^2^/g, micropore volume of 0.35 cm^3^/g, and pore width of 1.8 nm.

**Table 1:** The composition and characteristics of raw corn stover. [^a^Dry basis, ^b^Calculated by difference, ^c^Dry and ash-free basis].

Property	Value
**Proximate analysis (wt%)**^**a**^	
Moisture content	5.12 ± 3%
Volatile matter	77.49 ± 5%
Ash	6.16 ± 2%
Fixed carbon^b^	11.32 ± 3%
**Ultimate analysis (wt%)**^**c**^	
C	44.47 ± 2%
N	0.97 ± 1%
H	6.19 ± 2%
S	0.48 ± 1%
O^b^	47.88 ± 3%
Bulk density (kg/m^3^)	67.33 ± 2%
Higher heat value (MJ/kg)	16.71 ± 1%

### Experimental set-up and procedure

The current study used a modified domestic microwave oven (LG, 28 L convection oven) with a 1-L cylindrical quartz reactor. The diagram of the experimental set-up, as well as other essential information on the experimental set-up and procedures followed, are detailed in our previous work^28–30^. Based on our prior work^4^, the experiments were conducted under different microwave powers of 500, 700, and 900 W and different ratios of AC additive (10, 20, and 30%) with a constant reaction time of 15 min.

### Optimization of pyrolysis conditions for bio-oil and biochar yields

Response surface methodology (RSM) based on central composite design (CCD) is one of the most successful experimental designs for optimization of process parameters with the less possible number of experiments^31^. The interrelation between independent and dependent variables can be clearly known with this method. In this study, the optimized levels of microwave power (500 W, 700 W, and 900 W) and the ratios of AC as an additive (10%, 20%, 30%) were considered for investigation.

The effects of these parameters were investigated on the yields of bio-oil and biochar, the desirable products for this study. After knowing the optimal condition of pyrolysis for maximum yield of bio-oil followed by biochar, the physical and thermochemical properties of both products were evaluated and characterized. The independent variables were coded as microwave power (*X*_*1*_), and additive to biomass ratios (*X*_*2*_). Table 2 shows the details of the coded and actual values of the experimental design. The product yields of bio-oil and biochar were chosen as dependent variables. For statistical calculations, the variables *X*_*i*_ are coded as *x*_*i*_ according to Eq. (1).

1 $${x}_{i}=\left({X}_{i}-\frac{{x}_{0}}{\Delta x}\right)$$

where ${x}_{i}$, ${X}_{i}$, ${x}_{0}$, and $\Delta x$ are respectively the coded value for any independent variable, actual value, central value of the independent variable and step change. A 2^2^ factorial CCD and 5 (4 axials and one central) points and 3 replications were employed to optimize the pyrolysis conditions for maximum yields of bio-oil and biochar. The second-degree polynomials as per the Eq. (2) were calculated using the DESIGN EXPERT version 13 software statistical package (DOE 13, Stat-Ease, Minneapolis, MN, USA), (https://www.statease.com/software/design-expert/) to estimate the response of the dependent variables.

2 $${Y}_{i}= {a}_{1}+ {a}_{2}{X}_{1}+{a}_{3}{X}_{2}+{a}_{5}{X}_{1}{X}_{2}+{a}_{4}{X}_{1}^{2}+{a}_{6}{X}_{2}^{2}$$

where the parameters ${a}_{1}, {a}_{2},{a}_{3},{a}_{4},{a}_{5}, \mathrm{and} {a}_{6}$ are regression coefficients, while ${Y}_{i}$, ${X}_{1}, and {X}_{2}$ are predicted response and the independent variables microwave power and additive to biomass ratios respectively. The experimental data from the results of the model were analyzed statistically and the fitness of the predicted model was checked by the R^2^ value for providing the validation percentage of the model. Furthermore, the statistical significance of the independent parameters, considered in the study, was performed by the F-test method using a one-way analysis of variance (ANOVA) table at a significance level of 0.05 (p-value ≤ 0.05).

**Table 2:** Coded and actual values for the experimental ranges of independent variables.

Variables	Coded	Range and levels		
		− 1	0	+ 1
Microwave power (W)	*X*_*1*_	500	700	900
Additive to biomass ratio (%)	*X*_*2*_	10	20	30

### Product characteristics

The proximate analysis determines the components such as fixed carbon, volatile matter, moisture content and ash content present in feedstock and biochar for assessing the combustible and non-combustible constituents present among them. Ultimate analysis was done to determine the elemental composition such as CHNS of biomass or its pyrolytic products. The elemental analyses were conducted using Elementar (Germany), model UNICUBE CHNS/O analyser as per standards ASTM D 5291 and ASTM D1552. The Higher heat value (HHV) for raw CS and its biochar were measured by bomb calorimeter, while for bio-oil, it was calculated with the help of standard equation^32^.

The water content in the bio-oil was measured using VEEGO/MATIC-D Volumetric Karl Fischer titration according to ASTM E203. The acidity, dynamic viscosity, and density of bio-oil were also measured at 25 °C. The chemical compositions of bio-oil samples were identified by gas chromatography-mass spectrometer (GC–MS) (Agilent 5890–5973). There is the provision of HP-5 capillary column in the system. The carrier gas was helium (99.999% purity) with constant flow rate of 1.2 mL/min. The volume of injected sample (1:20 split ratio) was 1 µL. The temperature of the oven was initially set at 40 °C for 40 min in the system program, then it was raised by 5 °C/min to 250 °C and held for 5 min. The injected and detector temperatures were maintained at 250 °C and 230 °C, respectively. Different compounds present in the bio-oil sample were identified by comparing their mass spectra with standard mass spectra data library of National Institute of Standards and Technology (NIST).

### Techno-economic and environmental assessment

#### Technoeconomic analysis

The parameters considered for estimating the techno-economic assessment of the practice followed in this study include the initial cost of the experimental set-up; components used in the solar photovoltaic (PV) system, and the cost for life cycle analysis of the system. This assessment is evaluated in the scale-up system using grid electricity and solar PV power. The scale-up system is expected to process approximately 1125 tonnes of feedstock per year^33^. This requires about a 150-kW microwave reactor to process 750 kg of biomass feedstock per batch^34^ with one h duration for completion^33^ of the process and performing 5 batches for 10 working hours in a day^13^. In addition, the pyrolysis activities can be undertaken for 300 days per year. The practice, therefore, requires four labourers to be engaged per day to prepare the feedstock and operate the system. The parameters considered to estimate the techno-economic assessment of the MAP system are shown in Table 3.

**Table 3:** Description of operating conditions of scale-up microwave pyrolysis production system. [^a^Capacity for scale-up system, ^b^Total duration of the batch includes preparing the sample, reaction time, cooling down, and collect the products, ^c^Annual operating time considering, ^d^Based on https://tradingeconomics.com/egypt/minimum-wages, ^e^FAO, 2017].

Description	Unit	Scale-up production system	References
**Operating conditions**			
1. System capacity^a^	kg/batch	750	
2. Operation time	h/day	10	^13^
3. Total duration per batch^b^	h	2	
4. Number of batches per day	Batch/day	5	
5. Annual operating time^c^	h/year	300	
6. Feedstock	tonnes/year	1125	
7. Number of labors	–	4	
8. Operating time of the labors	h/year	12,000	
**Operating cost measurements**			
1. Transportation of feedstock to project site and pyrolytic products to market	%	2% of capital cost	
2. Maintenance	%	4% of capital cost	^33^
3. Insurance and taxes	%	1.5% of capital cost	
4. Consumables	%	4.5% of capital cost	
5. Other miscellaneous expenses	%	2% of capital cost	
Labour cost^d^	USD/year	Number of operating hours per year × wage cost per hour (UAD1.2/h)	
Feedstock cost^e^	USD/year	Amount of feedstock (tonne) per year × cost of the feedstock per unit (USD40/tonne)	^1^

#### Environmental assessment

The environmental aspect, in the form of mitigating the emissions of CO_2_ and earning the carbon credit potential through the scale-up MAP system, has been assessed. For the calculation of the quantity of CO_2_ emissions, it has been reported that about 1.5 kg of CO_2_ is emitted into the environment by burning 1 kg of CS feedstock^1,2^. This quantity of CO_2_ can be mitigated by applying the MAP process for the safe disposal of agricultural wastes like CS^28,29^. In addition, the coal-based thermal power plant generally produces about 0.98 kg of CO_2_/kWh. Taking distribution and transmission losses into account, a thermal power plant based on coal materials is expected to emit about 1.57 kg of CO_2_/kWh^35^. This quantity of CO_2_ emissions can also be mitigated by integrating solar PV electricity, clean energy, with the microwave pyrolysis practice.

## Results and discussion

### Response surface analysis

**Figure 2:**
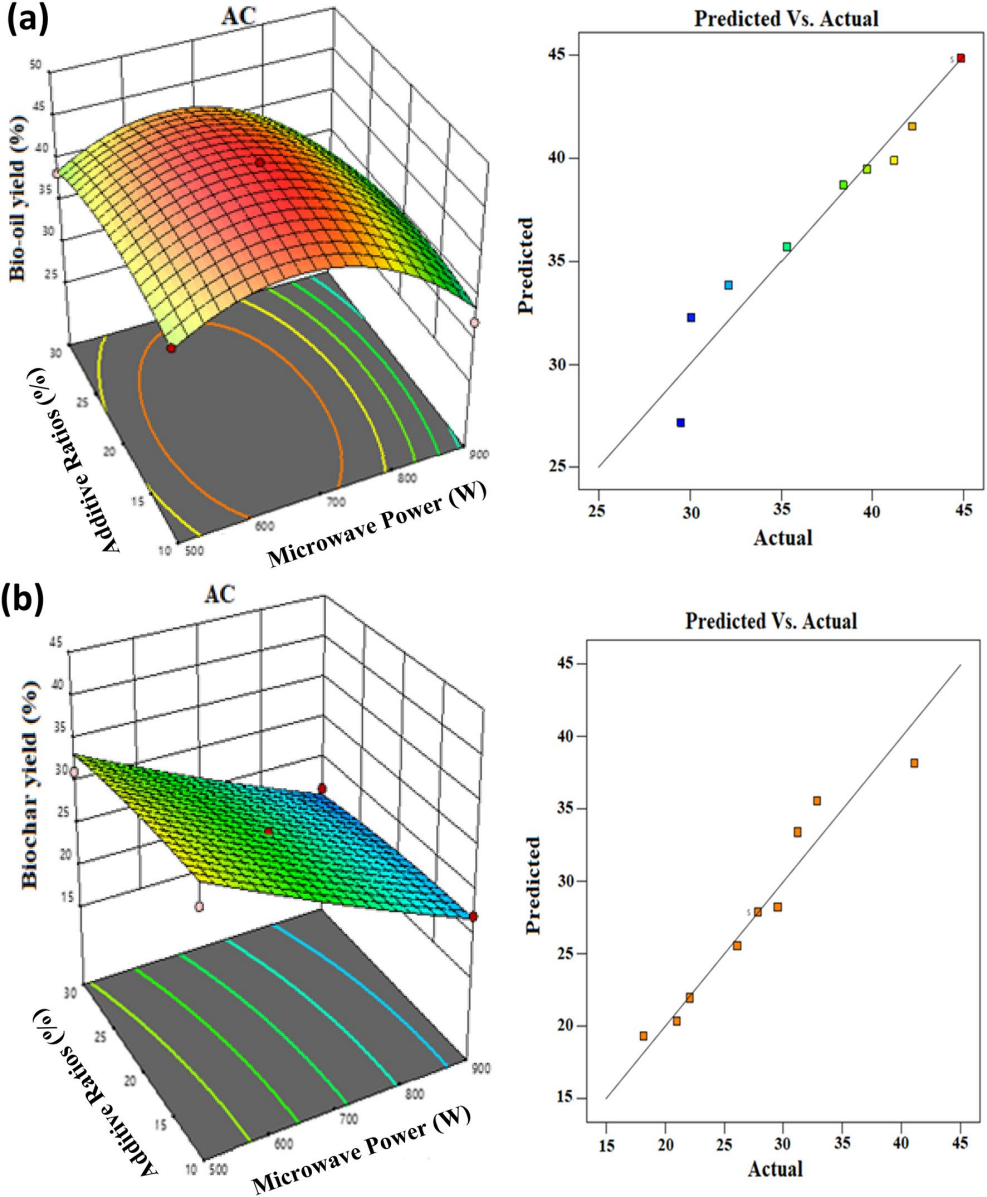
Response surfaces analysis of the products; yield (left) and predicted versus actual responses (right) for (**a**) bio-oil and (**b**) biochar.

Figure 2 shows the influence of quadratic terms of the dependent parameters on bio-oil and biochar yields along with predicted versus actual responses for each product when using AC as an additive. The quadratic model equations for response Y_Bio-oil_ and response Y_biochar_ were obtained using the results of the experiments and presented in Eqs. (3) and (4), respectively. The relation between predicted versus actual responses for bio-oil and biochar when using AC as an additive were presented in Fig. 2a (right) and 2b (right) respectively.

3 $${Y}_{Bio-oil}=-35.24+0.221735 {X}_{1}+0.835176 {X}_{2}-0.000095 {X}_{1}{X}_{2}-0.000168 {X}_{1}^{2}-0.020694 {X}_{2}^{2}$$

4 $${Y}_{Biochar}=57.18-0.049284 {X}_{1}+0.056719 {X}_{2}+0.000071 {X}_{1}{X}_{2}+0.00001 {X}_{1}^{2}-0.005037 {X}_{2}^{2}$$

From Fig. 2, it can be seen that the actual values are relatively distributed close to the linear line, indicating a high predictive value for the dependent variable under the studied range. Table A-1 in the Supplementary materials Appendix A presents the analysis of variance (ANOVA) for optimization of bio-oil and biochar yields using AC additive. From Table A-1, the ANOVA results, the P-values for bio-oil (< 0.0001) and for biochar (0.0004) were less than 0.05, suggesting that these equations for the resulted models were statistically significant at a 95.0% confidence interval (p < 0.05) to describe the yields of bio-oil and biochar. The coefficients of determination (R^2^) for the bio-oil and biochar response (Eqs. 3 and 4) were found to be 0.97 and 0.95 respectively. This implies that microwave power and different AC additive to biomass ratios under the studied ranges had a significant catalytic influence on bio-oil and biochar distribution yields during MAP of CS.

### Products yield and their characteristics

The products, yield and their characteristics have been evaluated under optimal pyrolysis condition (700 W and 10% AC). The optimal condition has been established from the optimization process with the aim of maximum bio-oil yield followed by biochar (the desirable products of the study). Table 4 shows the main properties of biochar and bio-oil from microwave pyrolysis of CS under optimal pyrolysis condition. Under optimal condition, the yields of the products were found to be 45 wt%, 28 wt%, and 27 wt% for bio-oil, biochar, and gas, respectively (Table 4). This means, under optimal pyrolysis condition, the bio-oil and biochar yields represent about 74 wt% of the total weight of raw CS used in the experiments. This clearly indicates that the major percentage of raw CS feedstock is converted into solid and liquid products as compared to the gas product (26 wt%). The comparison between the product yields under different pyrolysis condition can be found in the supplementary materials in Appendix A Table A-2. The biochar with HHV of 26 MJ/kg was achieved compared to 16.7 MJ/kg for raw CS and can be used as a solid biofuel in many applications.

**Table 4:** Main properties of biochar and bio-oil from microwave pyrolysis of corn stover with AC. [^a^Dry basis, ^b^Calculated by difference, ^c^Dry and ash-free basis, ^d^Density and viscosity at 25 °C, ^e^Calculated by HHV (MJ/kg) = 0.3382 C% + 1.4428 (H% − 0.125 O%)].

Biochar characteristics		Bio-oil characteristics	
Property	Value	Property	Value
**Proximate analysis (wt%)**^**a**^			
Volatile matter	30.79 ± 5%	pH value	5.27 ± 3%
Ash	13.18 ± 3%	Dynamic viscosity (mPa.s)^d^	2.89 ± 5%
Fixed carbon^b^	56.03 ± 3%	Water content (%)	38.92 ± 6%
**Ultimate analysis (wt%)**^**c**^		**Ultimate analysis (wt%)**^**c**^	
C	71.10 ± 7%	C	31.15 ± 1%
N	0.2 ± 3%	N	0
H	2.7 ± 7%	H	11.24 ± 2%
S	0	S	0
O^b^	26 ± 9%	O^b^	57.61 ± 2%
Bulk density (kg/m^3^)	33 ± 3%	Density (g/mL)^d^	1.036 ± 1%
HHV (MJ/kg)	25.79 ± 3%	HHV (MJ/kg)^e^	16.36 ± 3%
Yield (wt%)	29 ± 2%	Yield (wt%)	45 ± 4%
Energy yield (%)	43.45 ± 2%	Energy yield (%)	43.29 ± 3%

In addition, the energy yield from biochar with respect to the energy content in the raw CS was about 44%. Also, the biochar produced from the present study contains low content of nitrogen and was free of sulphur compared with raw CS biomass. This indicates that the biochar produced can be utilised as an environmentally friendly and clean solid biofuel to replace conventional coal, reducing greenhouse gas emissions such as CO and CO_2_, as well as harmful gas emissions such as SOx and NOx into the atmosphere.

For bio-oil, Table 4 shows that the HHV and energy yield from bio-oil reached 16.4 MJ/kg and 44% respectively which is due to the higher yield of bio-oil. Also, the bio-oil free from sulphur and nitrogen contents represents a beneficial feature for its use as a cleaner biofuel with respect to reduced emissions of greenhouse gases to the atmosphere. The moderate values of pH (5.3), viscosity (2.89 mPa s), and water content of 38.92% of the bio-oil were also achieved. The GC–MS analysis of the bio-oil revealed that the chemical composition was classified into 9 groups as well as the unidentified compounds (as per a match factor of 85%). These groups are acids, phenols, furans, guaiacols, esters, alcohols, hydrocarbons, ketones/aldehydes, and nitrogenous compounds. The contents of hydrocarbons and phenols in the bio-oil were up to 36% and 28% respectively with low oxygen-containing compounds (2%), low acids (2%), esters (1.2%), and alcohols (2%), in addition to furans content of 6% and ketones/aldehydes of 7%. The details of the bio-oil chemical composition analysed by GC–MS can be found in the Supplementary Materials Appendix A Table A-3. As a result of the increased quality of both bio-oil and biochar, produced from this study, both the products can be considered as a source of biofuel in many applications.

### Techno-economic environmental assessment

#### Tecno-economic assessment

For scale-up system, it is reported that a 150 kW microwave reactor can process 750 kg of feedstock per batch^34^ with 1 h duration for completion^33^ of the process and performing 5 batches for 10 working hours in a day^13^. The pyrolysis activities can be undertaken for 300 days per year. The electricity required to operate the system was calculated to be 780 kWh per day [(150 kW microwave reactor + 4 kW water pump + 2 kW temperature monitoring set-up) × reaction time per batch (1 h) × number of batches per day (5)]. In the case of operating the system by solar PV system, the required PV system should be sized accordingly. The sizing and cost estimation of solar PV system for both the systems have been presented in the Table 5 as per the calculations and explained in supplementary materials Appendix B.

**Table 5:** Sizing and cost estimation of solar PV system. [^a^Cost was calculated based on Egyptian exchange rate, ^b^Wiring and connections 5% of the sum of solar module, charge controller, batteries and inverter^35^].

Component	Cost per unit (USD/unit)^a^	Scale-up solar-powered production system		
		Number	Sizing	Total cost (USD)
1. Solar module	0.47/W_p_	780	300 W_p_	109,980
2. Charge controller	0.86/A	195	50 A × 24 V	8385
3. Batteries	0.59/Ah	2490	300 Ah × 12 V	440,730
4. Inverter	0.14/W	26	10,000 W	36,400
5. Wiring and connections^b^				29,775
Total				625,270

Table 6 illustrates the estimation of total expenditures with respect to capital and operating costs as well as the revenues to be earned from both the production systems. The capital cost has been estimated based on the studies carried out by the previous researcher in microwave pyrolysis^33^ considering a 3% escalation factor per year for the price of the components. Operating costs, such as feedstock and pyrolytic product transportation, maintenance, and other miscellaneous costs, are estimated as percentages of the capital cost (see Table 3). The cost of the electricity required to operate the system (in case of grid electricity used) has also been calculated.

**Table 6:** Techno-economic and environmental assessment for scaling-up microwave pyrolysis production system of corn stover. [^a^Based on Table 5, ^b^Total cost of the microwave pyrolysis system include the reactor, microwave system, condensation system, N_2_ gas, and temperature monitoring system was estimated based on previous work in microwave pyrolysis^33^, considering 3% escalation factor per year (https://fred.stlouisfed.org/), [200000 + (200,000 × 3/100 × 12 year)] = USD272000), ^c^Annual yield of the biochar was 28 wt% from annual feedstock consumed (1125 tonne/year × 28 wt%/100 = 315 tone biochar/year), ^d^Annual yield of the bio-oil was 45 wt% from annual feedstock consumed (1,125,000 × 45 wt%/100 = 506,250 kg bio-oil/year). Considering the density of the bio-oil is 1.036 g/mL (~ 1 g/mL) Table 4, thus the annual bio-oil yield was estimated to be 506,250 L/year, ^e^Selling price of biochar as fertilizer or soil conditioner and fuel in domestic cooking was estimated to be USD500/tonne, ^f^Selling price of bio-oil for chemical and phenol extraction was estimated to be USD1/L, ^g^Net annual income is equal to total annual revenues—total operation cost].

Item	Unit	Production system	
		Solar-powered system	Grid electricity system
System capacity	kg/batch	750	750
**Capital cost**			
1. Solar PV system	USD	625270^a^	–
2. Microwave reactor system^b^	USD	272,000	272,000
3. Total capital cost	USD	897,270	272,000
**Operating cost**			
1. Feedstock	USD/year	45,000	45,000
2. Labour	USD/year	14,400	14,400
3. Electricity	USD/year	-	18,720
4. AC additive	USD/year	2160	2160
5. Maintenance	USD/year	35,890	10,880
6. Transportation	USD/year	17,945	5440
7. Consumables	USD/year	40,377	12,240
8. Insurance and taxes	USD/year	13,459	4080
9. Other miscellaneous expenses	USD/year	17,945	5440
10. Total operation cost	USD/year	187,176	118,360
**Annual products yield**			
1. Biochar^c^	Tonne/year	315	315
2. Bio-oil^d^	Liter/year	506,250	506,250
**Annual revenues**			
1. Biochar^e^	USD/year	157,500	157,500
2. Bio-oil^f^	USD/year	506,250	506,250
Total annual revenues	USD/year	663,750	663,750
Net annual income^g^	USD/year	476,574	545,390
Payback period	Year	1.9	0.5
Monthly income	USD/month	39,700	45,400
**Environmental assessment**			
1. CO_2_ mitigation	Tonne/life	20,549	16,875
2. Credit earned from CO_2_ mitigation	USD/life	51,373	42,187

The cost of the electricity, when using grid electricity, was estimated to be USD18720 per year (780 kWh per day × USD0.08/kWh (Rs6/kWh) × 300 working days/year). Feedstock cost is estimated to be USD40 per tonne^1^. The proposed scale-up system is assumed to process about 1125 tonnes (750 kg per batch × 5 batches/day × 300 working days/year) as seen in Table 3. Percentages in the yield of pyrolytic products were estimated to be 45 wt%, 28 wt%, and 27 wt% for bio-oil, biochar, and pyrolytic gas, respectively, as per our experimental condition when pyrolyzed at 700 W microwave power and using AC additive as presented in Table 4 and Table A-2 in the “Supplementary materials S1”.

These pyrolysis conditions were selected as it produced the higher yields of bio-oil along with biochar (desirable products for the present study) with high quality. Also, the AC additive can be used as an additive repeatedly used in the MAP process system for 25 days^4,24^. The cost of AC is USD 2.4/kg and the required quantity per batch is 75 kg (750 kg feedstock/batch × 10/100 ratio of AC).

Thus, the total cost of using AC was estimated to be USD180 per 25 days (75 kg AC × USD2.4/kg). The total cost of AC per year is estimated to be USD2160 per year (USD180 per 25 days × 300 working days per year). The revenues to be generated by trading the pyrolytic products i.e., bio-oil and biochar have been estimated, based on the current prevailing prices as per the Egyptian market, considering their quality achieved in this study. The bio-oil produced in this study has a high proportion of hydrocarbons (36%). Phenols compounds accounted for the second-highest proportion (28%). In addition, the bio-oil had a low concentration of guaiacols compounds (2%) and acids (2%). Thus, it is considered to be a good source for phenols and chemical extraction. Therefore, the cost of the bio-oil is estimated to be USD1 per kg and the density of the bio-oil is estimated to be 1.036 g/mL (~ 1 g/mL), thus price is USD1 per liter^33,36,37^. The annual bio-oil production is estimated to be 506,250 kg (liter) (1125 tonne feedstock per year × 1000 × 45 wt%/100 bio-oil yield).

For biochar, the selling price is estimated to be USD500/tonne^33,34,38^. The annual biochar production was estimated to be 315 tonne biochar per year (1125 tonne/year × 28 wt%/100 = 315 tone biochar/year) as per the optimal pyrolysis conditions.

#### Environmental assessment

The emission of CO_2_ can be mitigated by applying the MAP practice operated by solar PV electricity for more sustainable of reutilize the agriculture residues. As the result from the practice, the mitigation of CO_2_ emission during 10-year lifetime of the set-up was estimated to be 20,549 tone/life for solar-powered system out of the calculation from the following equation:

Mitigation of CO_2_ emission = 1.57 kg CO_2_ from coal based thermal power plant/kWh/1000 × *E*_*out*_ kWh/year × life of set-up) + (1.5 kg CO_2_ emitted per kg of agricultural residue burning × kg of feedstock pyrolyzed per year × life of set˗ up).

Whilst, in the case of grid electricity system, the mitigation of CO_2_ emission was estimated to be 16,875 tonne/life for grid electricity system out of the calculation from the following equation:

Mitigation of CO_2_ emission = 1.5 kg CO_2_ emitted per kg of agricultural residue burning × kg of feedstock pyrolyzed per year × life of set˗ up.

For the estimation of credit earning by mitigation of CO_2_, it is reported that about USD 2.5 per mitigating one tonne of CO_2_ is credit earned (www.ecx.eu). Therefore, the carbon credit earned from the mitigation of CO_2_ to the environment is estimated to be USD51373 per life and USD42187 per life for solar-powered and grid electricity respectively out of the calculation from the following equation:

Carbon credit earned = USD2.5 per tonne × CO_2_ emission mitigated by the set-up per life.

As the result from the techno-economic and environmental analyses, the scaling-up of the MAP system for treating the agro-residues can be economically and environmentally beneficial. It is assessed that by using grid electricity, the payback period would be very nominal (0.5 year) compared to using a solar-powered system (1.9 years), resulting into earning more profit from the set-up during the rest of the life period of the system. Similarly, the benefit–cost ratio would be higher, causing the production system to be more cost-effective. This is because of the higher capital cost in the case of the solar-powered system. However, the mitigation of CO_2_ emission during the lifetime of the set-up is assessed to be more 20,549 tonnes/life when using the solar-powered system compared to 16,875 tonnes/life by the grid electricity system. This results into more credit earned from carbon dioxide mitigation i.e. USD51373 per life for solar PV powered system and USD42187 per life for grid electricity powered system. The MAP system could be a serviceable practice for mitigation of emission of CO_2_, causing the effective utilization of agro-residues and increased sustenance for the environment.

## Conclusion

In the present study, the environmental and techno-economic aspects of biochar and bio-oil produced by the pyrolysis of corn stover using solar-powered microwave heating and activated carbon as an additive were investigated. The process is first optimized for producing maximum bio-oil yield, followed by biochar. The yields of both bio-oil and biochar were found to be maximal under optimal condition of 700 W microwave power and 10% of AC additive. The result showed that the cost-effective and environmentally sustainable integration of the microwave-assisted pyrolysis process with solar photovoltaic power to produce biofuel and value-added products is viable. In comparison to 16,875 tonnes of CO_2_ mitigation and USD 42,167 in carbon credit earnings from a grid electricity system, the solar-powered system may mitigate 20,549 tonnes of CO_2_ during the lifetime of the set-up, resulting in USD 51,373 in carbon credit earnings. This will assist to reduce the power required for the processes and eliminate the use of fossil-fuel-based electricity, resulting in additional advantages for users and a more environmentally friendly approach to the technology's long-term sustainability. The findings from the present study would ultimately provide appropriate information regarding the technical feasibility and economic viability of the practice for its acceptability among the users. Future research should also pay attention to more investigations on a large scale, making the process suitable for adoption among the producers and making it more beneficial.

## Supplementary Information

Supplementary Information.

## Data Availability

All data generated or analyzed during this study are included in this published article.
