# Supplementary material for: Process optimization and technoeconomic environmental assessment of biofuel produced by solar powered microwave pyrolysis
Source: Sci Rep. 2022 Jul 22;12:12572. doi: 10.1038/s41598-022-16171-w (PMC9307767; doi:10.1038/s41598-022-16171-w)
Supplement: Supplementary file 1 — Supplementary Information. [file 41598_2022_16171_MOESM1_ESM.docx]

**Supplementary Materials**

**Process optimization and technoeconomic environmental assessment of biofuel produced by solar powered microwave pyrolysis**

Ahmed Elsayed Mahmoud Fodah ^a,^*, Taha Abdelfattah Mohammed Abdelwahab ^a,^*

^a^ College of Agricultural Engineering, Al-Azhar University, Cairo 11765, Egypt

* Corresponding Authors: [ahmedfodah@azhar.edu.eg](mailto:ahmedfodah@azhar.edu.eg), tahaabdelfattah@Azhar.edu.eg

<https://orcid.org/0000-0002-2345-0288>, <https://orcid.org/0000-0002-4916-8117>

**Appendix A**

**Table A-1** Analysis of variance (ANOVA) for optimization of bio-oil and biochar yields using AC additive. [^a^: is Sum of squares, ^b^: is Degree of freedom, ^c^: is Mean square].

| Source | | SS^a^ | DF^b^ | MS^c^ | F-value |  | p-value | Remarks |
| --- | --- | --- | --- | --- | --- | --- | --- | --- |
|  | **Bio-oil** | | | | | | | |
| Model | | 399.18 | 5 | 79.84 | 35.35 |  | < 0.0001 | significant |
| *X_1_*- Microwave power | | 72.77 | 1 | 72.77 | 32.22 |  | 0.0008 | significant |
| *X_2_*- Additive ratios | | 2.79 | 1 | 2.79 | 1.24 |  | 0.3029 |  |
| *X_1_X_2_* | | 0.14 | 1 | 0.14 | 0.0639 |  | 0.8076 |  |
| $X_{1}^{2}$ | | 313.39 | 1 | 313.39 | 138.76 |  | < 0.0001 | significant |
| $X_{2}^{2}$ | | 29.79 | 1 | 29.79 | 13.19 |  | 0.0084 | significant |
| Residual | | 15.81 | 7 | 2.26 |  |  |  |  |
| Lack of Fit | | 15.81 | 3 | 5.27 |  |  |  |  |
| Pure Error | | 0.0000 | 4 | 0.0000 |  |  |  |  |
| Total | | 414.99 | 12 |  |  |  |  |  |
|  | **Biochar** | | | | | | | |
| Model | | 367.17 | 5 | 73.43 | 21.06 |  | 0.0004 | significant |
| *X_1_*- Microwave power | | 356.50 | 1 | 356.50 | 102.25 |  | < 0.0001 | significant |
| *X_2_*- Additive ratios | | 7.21 | 1 | 7.21 | 2.07 |  | 0.1937 |  |
| *X_1_X_2_* | | 0.08 | 1 | 0.08 | 0.0233 |  | 0.8830 |  |
| $X_{1}^{2}$ | | 1.19 | 1 | 1.19 | 0.3416 |  | 0.5773 |  |
| $X_{2}^{2}$ | | 1.77 | 1 | 1.77 | 0.5063 |  | 0.4998 |  |
| Residual | | 24.41 | 7 | 3.49 |  |  |  |  |
| Lack of Fit | | 24.41 | 3 | 8.14 |  |  |  |  |
| Pure Error | | 0.0000 | 4 | 0.0000 |  |  |  |  |
| Total | | 391.58 | 12 |  |  |  |  |  |

**Table A-2** Products distribution under different pyrolysis conditions.

| Additive | Microwave power (W) | Products distribution % | | |
| --- | --- | --- | --- | --- |
|  |  | Biochar | Bio-oil | Syngas |
| Without additive | 500 | 40.21 | 33.61 | 26.17 |
| 10% AC |  | 32.89 | 39.71 | 27.4 |
| 20% AC |  | 31.44 | 40.22 | 28.34 |
| 30% AC |  | 31.22 | 38.42 | 30.36 |
| Without additive | 700 | 36.67 | 34.29 | 29.03 |
| 10% AC |  | 28.15 | 44.21 | 27.64 |
| 20% AC |  | 27.87 | 44.15 | 27.28 |
| 30% AC |  | 27.13 | 42.14 | 30.73 |
| Without additive | 900 | 26.5 | 33.02 | 40.48 |
| 10% AC |  | 22.11 | 32.12 | 45.77 |
| 20% AC |  | 21.23 | 31.48 | 47.29 |
| 30% AC |  | 21.01 | 30.07 | 48.92 |

**Table A-3** Relative area (%) of the main chemical compounds present in bio-oils produced from CS.

| Categories | Compounds Name | Area (%) |
| --- | --- | --- |
|  |  | 10% AC |
| Acids |  | |
|  | Acetic acid | 0.25 |
|  | Oleic acid | 1.78 |
| Phenols |  | |
|  | 4-Methyl, phenol | 1.32 |
|  | Phenol, 3-methyl | 1.85 |
|  | Phenol, 2-methoxy | 5.14 |
|  | Phenol, 4-ethyl | 9.18 |
|  | Phenol | 10.37 |
| Furans |  | |
|  | Furan, 3-pentyl | 0.67 |
|  | Benzofuran, 4,7-dimethyl | 2.10 |
|  | Furfural | 3.37 |
| Guaiacols |  | |
|  | 2-Methoxy-4-vinylphenol | 1.31 |
|  | Benzene, 1,4-dimethoxy- | 0.83 |
| Esters |  | |
|  | n-Butyl myristate | 1.23 |
| Alcohols |  | |
|  | Cyclopropyl carbinol | 2.13 |
| Ketones |  | |
|  | 2,3-Butanedione | 0.78 |
|  | 2-Cyclopenten-1-one | 0.57 |
|  | 3-Methylcyclopentane-1,2-dione | 0.50 |
|  | 2-Cyclopenten-1-one, 2-methyl- | 0.27 |
| Aldehydes |  | |
|  | Succindialdehyde | 1.72 |
|  | Pentanal | 3.15 |
| Hydrocarbons | 5-Undecyne | 0.64 |
|  | 9-Octadecyne | 3.66 |
|  | 1,19-Eicosadiene | 2.19 |
|  | Neophytadiene | 3.17 |
|  | 5-Ethyl-1-nonene | 1.98 |
|  | Naphthalene, 1,3/6/7-dimethyl- | 2.66 |
|  | Biphenyl | 2.13 |
|  | Dodecane, 2-methyl- | 3.91 |
|  | Fluorene, 4/2/1-methyl- | 2.62 |
|  | 2-Tetradecene, (E)- | 1.24 |
|  | Anthracene | 2.13 |
|  | Cyclohexane, methyl- | 1.36 |
|  | Docosane | 1.27 |
|  | 1-Hexacosene | 0.57 |
|  | 6-Tetradecyne | 0.68 |
|  | Hexadecane | 2.64 |
|  | Cyclopropane, nonyl- | 2.45 |
|  | 1-Hexene, 3,3-dimethyl- | 1.18 |
| Others |  | |
|  | Unidentified compounds | 15 |

**Appendix B**

**Sizing of solar PV system**

***Determination of designed electrical load***

In order to meet the losses occurred in the components of the PV system, the designed load should be higher than the actual load. The designed load was determined as 780 kWh/day based on the factor of safety to be 1.5.

$Design electrical load= 1.5 \times electrical load$ (1)

The factor 1.5 accounts for losses in the PV system components and the uncertainty to anticipate daily electrical load of appliances and also accounts for all the system efficiencies, including wiring and interconnection losses as well as the efficiency of the battery charging and discharging cycles.

***Determination of PV array size (W_p_)***

A peak sun shine hour, specifically, is the hour during which the intensity of sunlight is supposed to be 1,000 watts per square meter. The day length may be longer, but peak sun shine hours are less than that. The average peak sunshine hours per day on the optimum tilted surface in Egypt is considered as 7.0 h/day. Considering the available peak watt of module as 200W_p_ in the market_,_ the number of modules for the purpose was 3, each of 200 *W_p_*.

$PV array size required \left( Wp \right)= \frac{Designed load \left( \frac{Wh}{day} \right)}{peak sunshine hours per day} (2)$

$$Number of PV modules \left( N \right)= \frac{PV array size \left( W_{p} \right)}{Peak watt of PV module \left( W_{p} \right)} (3)$$

***Determination of inverter size (W) and charge controller capacity (A)***

The input rating of the inverter should never be lower than the PV array rating. The maximum continuous input rating of the inverter (*W*) should be about 10% higher than the PV array size to allow safe and efficient operation of PV power system as reported by SECO. Hence, the inverter size can be determined using equation (4). The inverter converts the DC power to AC power for operating AC electrical load. The charge controller saves the battery from over charging and over discharging and feeds the right configuration of current and voltage to the load. The charge controller capacity can be determined based on the PV array size and the operating voltage (such as 24V) designed for battery bank using equation (5).

$$Inverter capacity \left( W \right)= 1.1\times PV array size \left( Wp \right) (4)$$

$Charge controller capacity \left( A \right)=\frac{PV array size \left( W_{p} \right)}{Battery bank design voltage \left( V \right)}$ (5)

*Determination of battery bank size (Ah).* In actual practice, the battery bank size (*Ah*) can be determined by using the equation (6).

$$Battery bank size (Ah) = Autonomy days\times\frac{Electrical load (\frac{Wh}{day})}{Battery voltage (V)} (6)$$

The number of autonomy days for the PV system is taken to be 3 to account for three consecutive cloudy days and to improve battery life with shallow depth of discharge.

$Number of batteries =\frac{battery bank size (Ah)}{Ampere hour capacity of each battery (Ah)} (7)$

As per equation (6), the battery bank size was determined to be 300 *Ah* and 12 V. Thus, 2490 number of 300 *Ah*, 12 *V* batteries were required.

**Experimental observations related to solar PV system components**

The observations of various electrical parameters, measured during the operation of solar PV system were utilized to evaluate its performance. The electrical parameters such as open circuit voltage (*V_OC_*), short circuit current (*I_SC_*), fill factor (*FF*), solar radiation (*I_T_*) (*W/m^2^*) were measured throughout the year 2020 by using the instrument namely solar PV analyser (SEAWARD PV 200). The output and input power of the inverter were measured by AC/DC multi meter. The hourly experimental observations were measured for typical clear days for a month and continuing throughout the year 2020 in the warm and humid climatic condition as per the following equations.

$$Conversion efficiency of module \left( \eta_{Module} \right)= \left[ \frac{FF \times\left( V_{OC} \times I_{SC} \right)}{\left( I_{T}\times A \right)} \right] \times100 \left( \% \right) (8)$$

Due to the increased cell temperatures of the module compared to standard test condition in open field situation and other environmental factors such as dust deposition and reduced solar intensity, the module efficiency is reduced by 0.2 % annually. Therefore, average value for the efficiencies during 1^st^ and last year has been considered i.e., $\eta_{module (average).}$*A* is the area of module (m^2^), $\eta is efficiency.$

$${Efficiency of DC/AC inverter (\eta}_{Inverter})= \left[ \frac{(Output power)}{(Input power)} \right] \times100 \left( \% \right) (9)$$

Overall conversion efficiency of solar PV system:

$$\eta_{system}= \left[ \eta_{module (average)}\times\eta_{battery} \times\eta_{inverter} \right] \times100 \left( \% \right) (10)$$

*Energy output of PV system.* Energy output from the PV system is usually cite-specific and variable. It depends on the solar radiation, ambient air temperatures and efficiency of the module in on-field condition. The output of the PV system has been calculated with the equations (11 and 12).

$Annual average insolation \left( \frac{\frac{kWh}{m^{2}}}{year} \right)= hourly average solar radiation\frac{\frac{kW}{m^{2}}}{day}\times peak sunshine hours per day \left( h \right)\times number of clear sunny days in a year (11)$

$$Annual electrical energy output, Eout = Insolation (kWh/m^{2}/year)\times system efficiency (12)$$

Alternatively, *E_out_* can also be calculated as per equation (13):

$$Eout (kWh/year) = Daily electrical load (kWh/day) \times Number of days of operation/year (13)$$

**Carbon Dioxide (CO_2_) emission, mitigation and carbon credit**

*CO_2_ emission.* On-farm burning of CS is now-a-days the most prevailing practice followed throughout Egypt causing the emission of CO_2_ to the environment. It has been reported that by burning 1 kg of CS, 1.5 kg CO_2_ is released to the environment.

*CO_2_ mitigation.* CO_2_ mitigation can be accomplished by the use of solar PV system and preventing on-farm burning of CS. The electricity is mostly generated in Egypt from the coal based thermal power plant. It has been reported that a coal based thermal power plant produces 0.98 kg of CO_2_/kWh. In Egyptian conditions, considering 40% and 20% losses of energy in transmission and distribution systems respectively, the amount of emission is 1.5 kg of CO_2_/kWh of electricity generated from coal thermal power plant.

${CO}_{2} emission mitigated \left( \frac{kg}{life} \right)= 1.57 \left( \frac{kg}{kWh} \right)\times E_{out} \left( \frac{kWh}{year} \right)\times life of system + 1.5 kg {CO}_{2} emitted per kg of CS burning\times kg of CS pyrolysed/year\times life of system (14)$

*Carbon credit earned.* One carbon credit represents the mitigation of 1 ton of CO_2_ emission. The emissions of CO_2_ due to various energy utilizations and generations pose a major contributing factor to the greenhouse effects. International treaties for mitigating emissions of greenhouse gases across the globe have therefore introduced carbon credit trading which is an administrative approach for controlling pollution by providing economic incentives for the efforts to reduce the emissions of pollutants. Carbon credits are therefore a tradable permit scheme prevailing among the countries included in the strategic planning of controlling the emissions of greenhouse gases. The amount of carbon credit earned in the present study due to the use of PV system and preventing CS burning can be calculated by the equation (15).

$Carbon credit earned = \$\frac{2.5}{ton}\times{CO}_{2} emission mitigated by the set up \left( \frac{tons}{life} \right)$(15)

The amount $ 2.5/ton represents the monetary value of mitigating one ton of CO_2_ emission. The price of carbon credit is as per European Climate Exchange ([www.ecx.eu](http://www.ecx.eu)).
